# Induction of autophagy promotes the growth of early preneoplastic rat liver nodules

**DOI:** 10.18632/oncotarget.6810

**Published:** 2015-12-31

**Authors:** Marta Anna Kowalik, Andrea Perra, Giovanna Maria Ledda-Columbano, Giuseppe Ippolito, Mauro Piacentini, Amedeo Columbano, Laura Falasca

**Affiliations:** ^1^ Department of Biomedical Sciences, University of Cagliari, Cagliari, Italy; ^2^ Department of Biology, University of Rome “Tor Vergata”, Rome, Italy; ^3^ National Institute for Infectious Disease, IRCCS “Lazzaro Spallanzani”, Rome, Italy

**Keywords:** preneoplastic nodules, autophagy, amiodarone, chloroquine, miR-224

## Abstract

Although inhibition of autophagy has been implicated in the onset and progression of cancer cells, it is still unclear whether its dysregulation at early stages of tumorigenesis plays an oncogenic or a tumor suppressor role. To address this question, we employed the Resistant-Hepatocyte rat model to study the very early stages of hepatocellular carcinoma (HCC) development. We detected a different autophagy-related gene expression and changes in the ultrastructural profile comparing the most aggressive preneoplastic lesions, namely those positive for the putative progenitor cell marker cytokeratin-19 (KRT-19) with the negative ones. The ultrastructural and immunohistochemical analyses of KRT-19-positive preneoplastic hepatocytes showed the presence of autophagic vacuoles which was associated with p62, Ambra1 and Beclin1 protein accumulation suggesting that a differential modulation of autophagy occurs at early stages of the oncogenesis in KRT-19-positive vs negative lesions. We observed an overall decrease of the autophagy-related genes transcripts and a strong up-regulation of miR-224 in the KRT-19-positive nodules. Interestingly, the treatment with the autophagy inducer, Amiodarone, caused a marked increase in the proliferation of KRT-19 positive preneoplastic lesions associated with a strong increase of their size; by contrast, Chloroquine, an inhibitor of the autophagic process, led to their reduction. These results show that autophagy modulation is a very early event in hepatocarcinogenesis and is restricted to a hepatocytes subset in the most aggressive preneoplastic lesions. Our findings highlight the induction of autophagy as a permissive condition favouring cancer progression indicating in its inhibition a therapeutic goal to interfere with the development of HCC.

## INTRODUCTION

The term autophagy identifies the basic self-degradative physiological process by which the cell removes worn-out or damaged components, such as protein aggregates, mitochondria, Endoplasmic Reticulum (ER), peroxisomes and intracellular pathogens [1-3]. Autophagy is evolutionarily conserved and involves the double-membrane sequestration and lysosomal breakdown of the cargo [1-3]. Although there is a basal autophagy in the cell, this process is further induced in response to stress, e.g. nutrient deprivation, hypoxia and pressure overload to catabolize cellular substrates and generate energy [4]. Defects in the autophagy machinery have been associated with the pathogenesis of the major human diseases including cancer [1,4]. Three major forms of autophagy have been described: micro-, macro- and chaperone-mediated autophagy, all involving the lysosomal breakdown of sequestered material [4,5]. The emerging role of macro-autophagy (from here on referred to as autophagy) has been the focus of recent cutting edge research and it is now accepted that it plays a critical role in both health and disease [1-3]. Autophagy is a multistep process involving its induction, the development of an isolation membrane, the completion and maturation of an ‘autophagosome’ containing cytosolic components for recycling and the ultimate fusion with a lysosome (forming the autophagolysosome) for degradation by lysosomal enzymes [1-3].

While oncogenic transformation and tumour development are associated with resistance to, or loss of, the apoptotic pathway, an increasingly number of experimental data suggest that autophagy imbalance plays an important role in tumorigenesis and is a relevant target for cancer therapy [1, 5-7]. Indeed, autophagy-deficient mice develop a large number of spontaneous tumours, supporting the idea that autophagy acts as tumor suppression mechanism [6-7]. Moreover, the heterozygous deletion in mice of essential autophagy genes such as Beclin1 and Ambra1 leads to high incidence of spontaneous tumours [8, 9]. The role played by Beclin 1 and Ambra1 as tumour suppressors is also evidenced by the identification of their binding partners, most of which are implicated in tumorigenesis, such as Bcl-2. The anti-apoptotic member of the bcl-2 family constitutively binds to Beclin1 and Ambra1 complex inhibiting autophagy induction [10]. Furthermore, overexpression of the positive regulator of Beclin1 UVRAG activates autophagy and suppresses tumour cell growth, whereas its down-regulation results in decreases autophagy levels and triggers uncontrolled cell proliferation [7, 8]. Finally, it has been recently published that Ambra1 regulates cell proliferation by facilitating the degradation of the proto-oncogene c-Myc by favouring its dephosphorylation by PP2A and thereby reducing the cell division rate [8].

Although the current view is that autophagy acts as a tumour suppressor process it is now also emerging that some established cancers require autophagy to survive, thus suggesting a pro-tumour autophagy activity. In keeping with this notion, the Ras-dependent tumorigenesis has been shown to be associated to autophagy induction [11, 12]. It is also now clear that the genotype of an individual tumor influences the autophagic function. For instance, in melanoma, BRAF has a direct effect on autophagy regulation. In fact, the overexpression of the wild type BRAF vs the mutated BRAFV600E in melanoma cells leads to increased basal levels of autophagy [13].

The paradox of autophagy acting as both a cell survival pathway and a tumour suppressor pathway is now being in some way reconciled; the current view is that while autophagy suppresses tumour growth at early stages of oncogenesis, it promotes growth in established tumors [6, 11, 12]. However, due to several controversial data the exact role of autophagy in the different steps of cancer development, especially in hepatocellular carcinoma (HCC), remains elusive [14-16].

HCC is the third cause of cancer-related deaths worldwide, and is characterized by poor prognosis and few treatment options [17]. The development of HCC is a multistep process. HCCs arise most frequently in the setting of chronic liver inflammation and fibrosis due to viral infection, metabolic injury, toxic insults, or autoimmune reactions. These tumors originate from premalignant lesions, ranging from dysplastic foci to dysplastic hepatocyte nodules that are often seen in damaged and cirrhotic livers, and are more proliferative than the surrounding parenchyma [18]. Since no effective treatment for HCC exists and, upon diagnosis, most patients with advanced disease have a remaining lifespan of only 4–6 months, it is critical to detect cellular and molecular changes taking place in preneoplastic lesions and identify biomarkers and molecular targets useful for an early diagnosis and for therapy.

Since the knowledge of molecular events occurring in early stages of HCC development is hampered by the difficulties in the histomorphologic distinction between non-malignant nodular lesions and early HCCs, animal models allowing the study of different stages of hepatocarcinogenesis, represent a very helpful tool to detect cellular and molecular alterations occurring in early preneoplastic stages. Therefore, in our present study, we employed the Resistant-Hepatocyte (R-H) model which allows dissecting the several steps of hepatocarcinogenesis and whose translational value has already been demonstrated [19-21], to investigate the role of autophagy in the early stages of HCC development.

## RESULTS

### Differential autophagy modulation takes place in early preneoplastic nodules

**Figure 1:**
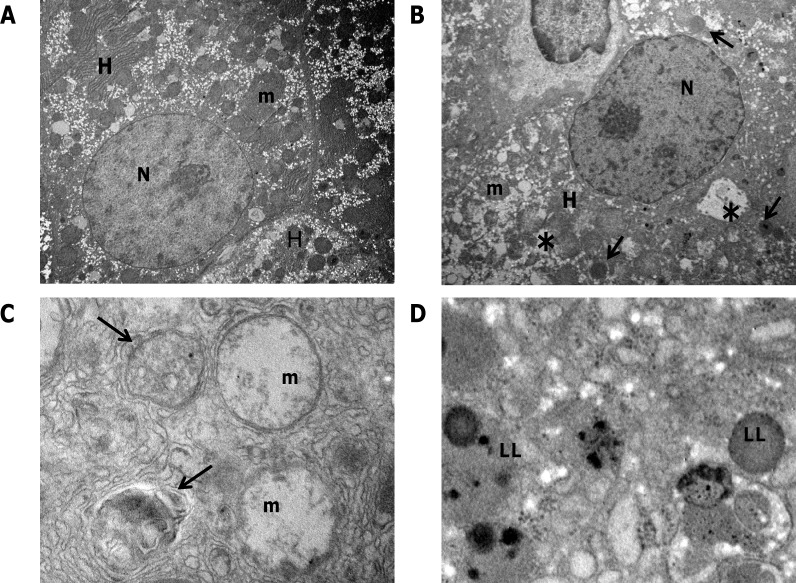
Transmission electron microscopy of preneoplastic nodules **A**. Representative image of control liver. Hepatocytes (H) typically display a large, round nucleus (N); numerous mitochondria (m) with regular matrix and prominent cristae. **B**. Ultrastructure of a preneoplastic hepatocyte (H). Irregular nucleus (N), extensive vacuolation (asterisks), lipid droplets accumulation (see arrows) characterize the preneoplastic modifications. **C**. Higher magnification of nodule hepatocytes revealed that mitochondria undergo pathological modifications, consisting of swelling and progressive loss of cristae. Autophagosomes containing only partially digested material are also visible (arrows). **D**. Lipid inclusions (LL) accumulation in the cytoplasm of preneoplastic hepatocytes. Original magnifications: A, B= x3000; C, D= x30000.

The R-H model allows dissecting the different steps of the carcinogenic process, as phenotypically distinct lesions can be identified at well-defined timings [19]. Therefore, it is possible to investigate changes in the autophagic machinery at very early times in HCC development. To this aim, we performed ultrastructural examination of preneoplastic nodules dissected from the surface of the liver exposed to the R-H model and developed 10 weeks after treatment with DENA. Electron microscopy of hepatocytes in the preneoplastic nodules revealed extensive morphological alterations of cytoplasmic organelles (Figure 1B) compared with control liver (Figure 1A). Most hepatocytes in the nodules displayed abnormal mitochondria (Figure 1C), which appeared swollen with loss of cristae and ruptures of the outer membrane. No sign of clearance by autophagy of the degenerating mitochondria was visible. Autophagic vacuoles containing only partially degraded materials (Figure 1C) and lipid droplets accumulation were frequently observed (Figure 1D). All these features are indicative of an impairment of the autophagic process, demonstrating that derangement of the autophagic machinery occurs at very early stages of HCC development.

### Autophagy modulation in KRT-19^+^ negative vs positive preneoplastic nodules

**Figure 2:**
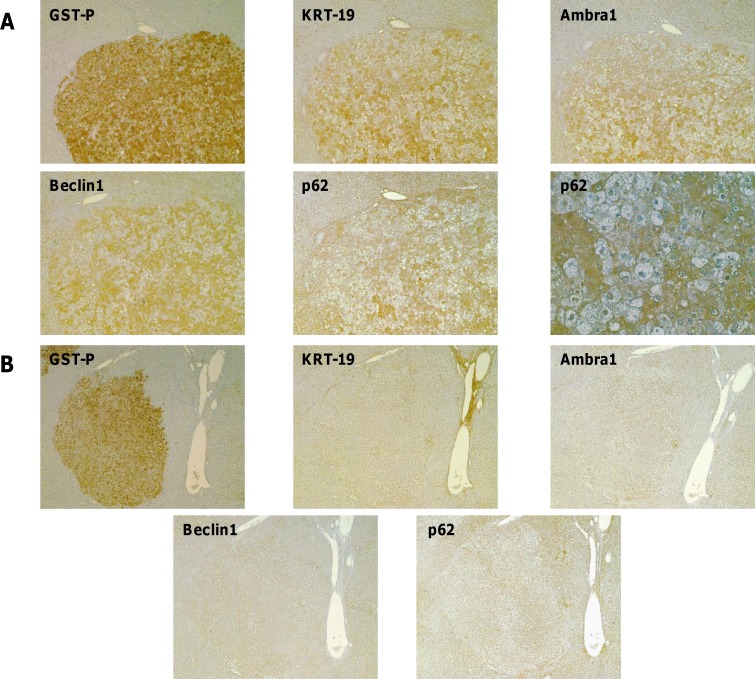
Autophagy impairment is restricted to a more aggressive subset of preneoplastic hepatic nodules IHC on serial sections of liver from rats subjected to the R-H protocol and sacrificed 10 weeks after DENA. **A**. Presence of intense staining for Ambra1, Beclin1 and p62 in a nodule positive for GST-P and KRT-19 (magnification x4). A higher enlargement of the same nodule showing nuclear p62 staining (magnification x20). **B**. Absence of Ambra1, Beclin1 and p62 staining in a GST-P^+^/KRT-19^−^ nodule (magnification x4).

We have previously shown in the R-H model [20,21] that although preneoplastic nodules positive for the putative progenitor cell marker cytokeratin-19 (KRT-19^+^) represent a minority of the total preneoplastic lesions, most HCCs are positive for this marker. This suggests that KRT-19^+^ preneoplastic nodules have an advantage in the progression to malignancy, while KRT-19^−^ lesions undergo spontaneous remodeling during the carcinogenic process, a previously described phenomenon [22]. Therefore, we wished to investigate as to whether autophagy modulation could be a general phenomenon of preneoplastic stages or it is restricted to the most aggressive (KRT-19^+^) lesions. To this aim, immunohistochemistry analysis of typical autophagic markers, such as p62, Ambra1 and Beclin1, that are involved in the multistep process of autophagy, was performed on serial sections of livers from rats subjected to the R-H protocol and sacrificed 10 weeks after DENA. Interestingly, specific staining of KRT-19+ - but not of KRT-19- -preneoplastic lesions was observed by positivity of the liver sections for two other proteins, such as Ambra1 and Beclin1, involved in the early regulatory steps of the autophagic process and p62 which is mediating the recruitment of cargos in the autophagosomes (Figure 2A, 2B). Remarkably, 99%, 94% and 71% of GST-P+/KRT-19+ nodules were also positive for Ambra1, p62 and Beclin1, respectively (Table 1). Almost none of KRT-19- nodules – nor the surrounding parenchymal tissue- exhibited staining for these proteins. As shown in Figure 2A and 2B, IHC revealed cytoplasmic and nuclear accumulation of p62 only in a subset of hepatocytes (about 50% of the total) in the GST-P^+^/KRT-19^+^ nodules, but not in GST-P^+^/KRT-19^−^ preneoplastic lesions, indicating that a marked differential modulation of the autophagic process occurs also inside the GST-P^+^/KRT-19^+^ nodules.

**Table 1:** Percentage and number of KRT-19^+^/Ambra1^+^, KRT-19^+^/p62^+^ and KRT-19^+^/Beclin1^+^ nodules with respect to the total amount of KRT-19+ nodules

Mean N° of GST-P^+^nodules	Mean N° of KRT 19^+^/GST-P^+^nodules	Mean N° of Ambra1^+^/KRT-19^+^nodules	% of Ambra1^+^/KRT-19^+^nodules	Mean N° of p62^+^/KRT-19^+^nodules	% of p62^+^/KRT-19^+^nodules	Mean N° of Beclin1^+^/KRT-19^+^nodules	% of Beclin1^+^/KRT-19^+^nodules
74.6 ± 13.3	18.4 ± 4.0	18.2 ± 4.0	99.0	17.3 ± 4.0	94.0	13.0 ± 2.2	70.6

The analysis of serial liver sections was performed on a total number of 373 nodules, obtained from 5 rats.

### Enhanced transcription is not the cause of the increased accumulation of p62, Ambra1 and Beclin1

**Figure 3:**
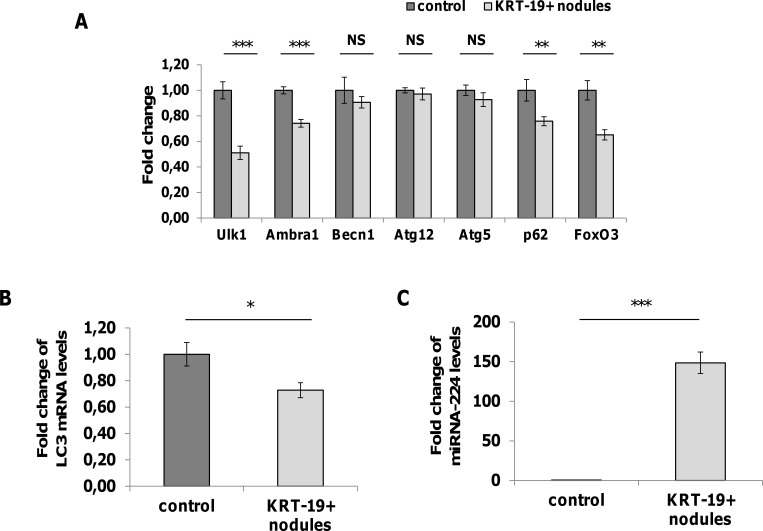
Analysis of mRNA levels of autophagy-associated genes and miRNA-224 **A**. qRT-PCR analysis of Ulk1, Ambra1, Beclin1, Atg12, Atg5, p62 and FoxO3 mRNA in KRT-19^+^ nodules generated 10 weeks after treatment with DENA. Gene expression is reported as fold-change relative to age-matched controls; ***P<0.001, **P<0.01, NS: not significant. **B**. LC3 expression in KRT-19+ preneoplastic nodules. Gene expression is reported as fold-change relative to age-matched controls; *P<0.05. **C**. qRT-PCR analysis of miR-224 expression in KRT-19^+^ nodules. miR-224 expression is reported as fold-change relative to age-matched controls; ***P<0.0001.

To assess whether the accumulation of p62, Ambra1 and Beclin1 is the consequence of transcriptional or post-transcriptional modifications of genes/gene products involved in the autophagic process, we performed qRT-PCR analysis of the expression of these and other autophagy-associated genes, on laser micro-dissected GST-P^+^/KRT-19^+^ preneoplastic nodules. As shown in Figure 3A and 3B, no increased transcription of any of the examined genes involved in the autophagic process was observed. Rather, we found down-regulation of some of them, such as Ulk1, Ambra1, p62, LC3 and FoxO3. These results clearly demonstrate that the accumulation of these proteins in aggressive preneoplastic lesions is not due to an active transcription. However, the interpretation of these findings should take into consideration that, as highlighted by the IHC, the accumulation of the Ambra1, p62 and Beclin1 has been detected only in about 50% of the hepatocytes.

### MiR-224 is highly expressed in KRT-19 positive nodules

Recently, it was reported that inhibition of autophagy in hepatitis B-associated human HCCs is responsible for the strong accumulation of miR-224 - one of the most up-regulated miRs in human cancer [23, 24] - in these tumors [25]. Therefore, we investigated the expression of miR-224 in KRT-19^+^ preneoplastic lesions. As shown in Figure 3C, miR-224 was strongly up-regulated in KRT-19 positive lesions (150-fold) compared to normal liver, supporting the notion that the increased miR-224 expression found in experimental and human HCC is most likely the consequence of its accumulation when autophagic machinery is deregulated.

### The autophagic inducer, Amiodarone, stimulates the growth of KRT-19^+^ nodules

**Figure 4:**
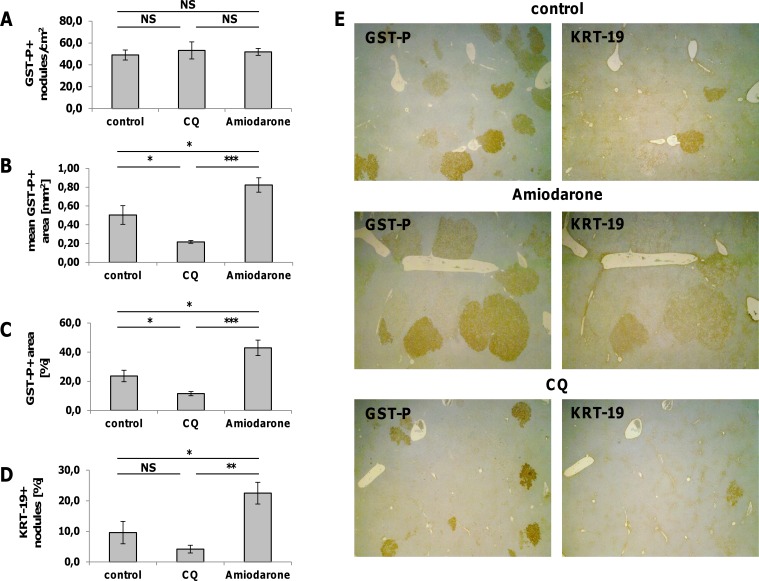
Effect of Amiodarone and Chloroquine on the number of GST-P^+^ nodules, the mean GST-P^+^ area, the percentage of the area occupied by GST-P^+^ hepatocytes and the number of KRT-19^+^ nodules Rats exposed to the R-H protocol were injected intraperitoneally with Amiodarone (30 mg/kg, four doses) or Chloroquine (CQ, 50 mg/kg, 4 doses) starting 2 weeks after 2-AAF withdrawal (6 weeks after treatment with DENA). Animals were euthanized 7 days after the treatment. Effect of Amiodarone and Chloroquine administration on the number of GSTP-positive nodules **A**., the mean GST-P-positive area **B**., the percentage of the area occupied by GST-P-positive hepatocytes **C**. and the number of KRT-19^+^ nodules **D**. Values are expressed as mean ± SEM. ***P<0.001, **P<0.01, *P<0.05, NS: not significant. **E**. GST-P and KRT-19 immunohistochemistry of liver sections from rats treated with Amiodarone and CQ (magnification x1.25).

**Figure 5:**
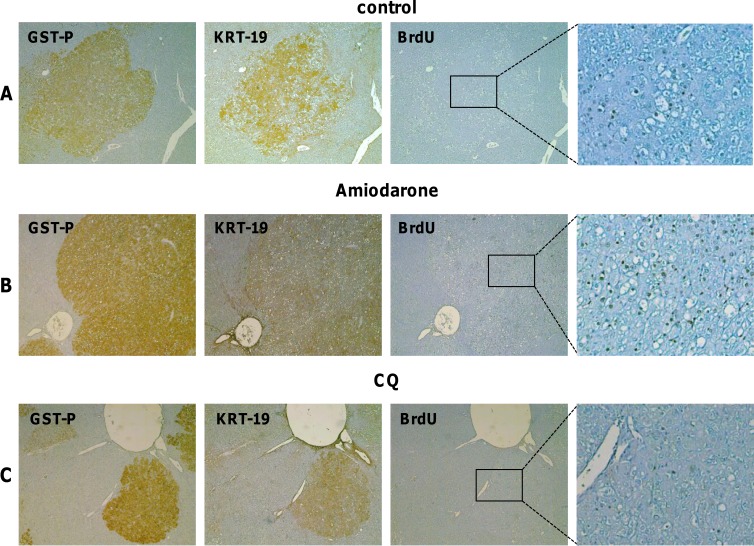
Effect of Amiodarone and Chloroquine administration on hepatocyte proliferation **A**. GST-P, KRT-19 and BrdU IHC on serial sections of liver from rats subjected to the R-H protocol and sacrificed 7 weeks after DENA (magnification x4, inset x10). **B**. GST-P, KRT-19 and BrdU IHC on serial sections of liver from rats subjected to the R-H protocol, treated with four doses of Amiodarone and sacrificed 7 days after treatment (magnification x4, inset x10). **C**. GST-P, KRT-19 and BrdU IHC on serial sections of liver from rats subjected to the R-H protocol, treated with four doses of Chloroquine (CQ) and sacrificed 7 days after treatments (magnification x4, inset x10).

The results so far obtained demonstrate that autophagy deregulation is an early event in HCC development and characterizes about 50% of the parenchymal cells in the most aggressive lesions. Since previous works suggested that autophagy mainly contributes to tumor suppression during the early stage of tumorigenesis, we wished to further investigate whether impairment of the autophagic process favours cancer progression. To address this question, we administered Amiodarone, an autophagy inducer [26], or chloroquine (CQ), an autophagy inhibitor [27], to nodule-bearing rats developed 6 weeks after DENA. Interestingly, while Amiodarone did not significantly modify the number of GST-P^+^ preneoplastic lesions (Figure 4A), an impressive increase of the size (Figure 4B, 4E) and of the % hepatic area (Figure 4C, 4E) occupied by these lesions was observed compared to the control group. These enhancing effects were associated with a strong increase in nodule hepatocyte proliferation (Figure 5A, 5B). Interestingly, Amiodarone also led to an increase of the percentage of KRT-19^+^ nodules (Figure 4D, 4E). On the opposite, when compared to untreated animals, the autophagic inhibitor CQ caused a decrease of the size and of the % hepatic area occupied by these lesions (Figure 4A-4C, 4E). Moreover, the % of KRT-19^+^ nodules was found to be significantly lower than that of the Amiodarone-treated group. Remarkably, treatment with CQ strongly inhibited proliferation of nodule hepatocytes (Figure 5C) when compared to either control- or Amiodarone-treated group.

**Figure 6:**
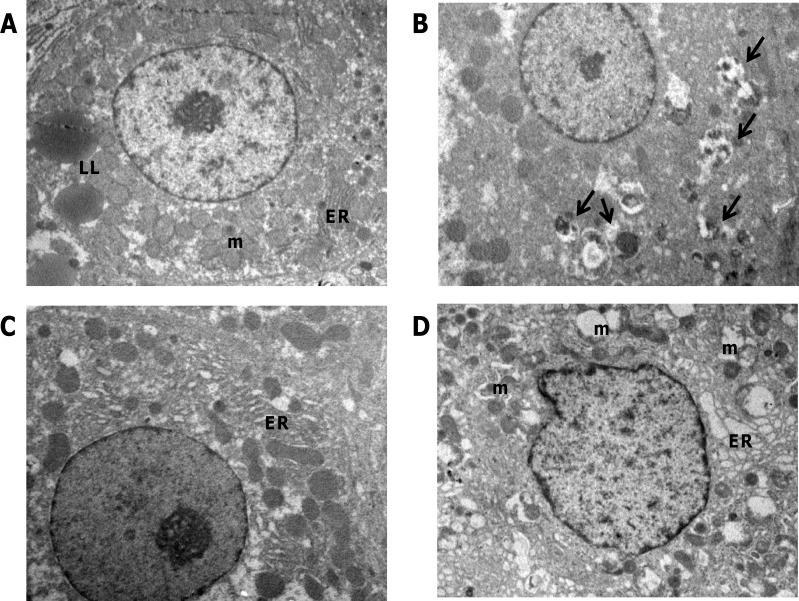
Ultrastructure of livers from nodule-bearing rats receiving amiodarone and chloroquine **A**. TEM examination of liver tissues from rats receiving amiodarone show that endoplasmic reticulum (ER) appears well preserved and mitochondria (m) swelling and vacuolation are reduced compared to untreated preneoplastic liver **D**.; Few lipid inclusions (LL) are displayed by these hepatocytes compared to control liver **C**. **B**. Massive accumulation of autophagic vesicles is observed following treatment with chloroquine. Original magnifications: x3000.

Previous report [25] has shown that Amiodarone-induced autophagy leads to a reduction of the tumor weight. Therefore, we wished to establish if the observed promoting effects on nodule growth induced by Amiodarone was associated with increased autophagy. To this aim, electron microscopy examination of nodules scooped out from the liver surface was performed. As shown in (Figure 6A), the fine structure of Amiodarone-treated hepatocytes revealed a less damaged morphology; lipid droplets scattered in the cytoplasm and dilated intercellular space were observed; however, after Amiodarone treatment cells in the nodules displayed intact rough endoplasmic reticulum and mitochondria which were similar to that of normal untreated hepatocytes (Figure 6C). Electron microscopy was also performed after CQ treatment; since the small size of CQ-treated rat nodules hampered the possibility of dissecting them from the liver, electron microscopy examination was performed in randomly selected areas of the liver. As shown in Figure 6B, EM analysis confirmed the inhibitory effect of CQ on autophagy; indeed, many vacuoles containing undigested cytoplasmic materials were observed. In addition, apoptotic and necrotic hepatocytes were often observed (data not shown). These findings indicate that while induction of autophagy stimulates the growth of preneoplastic lesions to HCC progression, inhibition of autophagy exerts the opposite effect.

## DISCUSSION

Recent years have brought an enormous advance in the elucidation of the molecular machinery involved in mammalian autophagy [1-3]. Although the contribution of autophagy in tumour development is unquestioned, nonetheless, its role is not completely understood. Paradoxically, autophagy seems to play a dual role, acting as tumor promoter or tumor suppressor, depending on the tumor stage [28]. Numerous studies suggest that autophagy contributes to tumor suppression during the early stages of tumorigenesis [28-29]. This suppressive effect is likely due to some important functions of autophagy, such as defective organelle elimination in order to reduce oxidative stress and prevent DNA damage. During the late phases of tumorigenesis, autophagy is supposed to act as tumor promoter mechanism, enabling tumor cells to cope with high metabolic demand [6, 11].

As far as the role of autophagy in HCC development is concerned, it was reported that homozygous deficient mice for the autophagy genes ATG5 or ATG7 develop benign liver tumors that do not progress to metastatic cancer, which suggests that loss of autophagy may be sufficient for the initiation of tumorigenesis, but residual autophagy is required for the progression to cancer [6]. In line with these results, it was also reported that autophagy suppressed hepatocarcinogenesis at early stages by protecting normal cell stability and promoted hepatocarcinogenesis at late stages by supporting tumor cells growth [28, 29]. Finally, Ambra1 haplodeficiency in mice leads to the development of liver tumors, and, in humans, Beclin1 expression was decreased in HCC tissues compared with adjacent non-tumoral tissues and Ambra1 [4, 5].

In this study, we attempted to investigate the status of the autophagic process in the very early stages of hepatocarcinogenesis, taking advantage of the well-established R-H model in the rat. The main findings stemming from our work indicate that modulation of autophagy i) is a very early event in the multistage process of hepatocarcinogenesis, and, ii) takes place selectively in in KRT-19^+^ preneoplastic lesions, but not in KRT-19^−^ lesions. We have previously shown that although KRT-19^+^ lesions represent a minority of the total preneoplastic lesions, most HCCs arising in this model are KRT-19^+^[20, 21]. Therefore, our results are in line with the observation that autophagy defects favour cancer development and loss of autophagy may increase the propensity of cells toward oncogenic transformation [30, 31].

Another relevant finding of the present work is the association between high miR-224 expression and autophagy impairment. A recent study demonstrated that inhibition of autophagy in HBV-associated human HCCs is the mechanism responsible for the strong accumulation of the mature form miR-224 in these tumors [25]. These results were also confirmed in liver tumors of HBV X gene transgenic mice. Importantly, miRNA-224, involved in cell proliferation, migration and invasion [32], is one of the most up-regulated miRs in rodent and human HCC [21-23, 24]. Indeed, our data show a drastic upregulation of miRNA-224 in those subset of nodules which are more aggressive and develop HCC.

An additional and novel finding of the present study is that while the autophagic inducer, Amiodarone, promotes a striking increase in the proliferation of nodular hepatocytes and the size of preneoplastic lesions, the inhibitor of autophagy, CQ, induces the opposite, thus leading to a decrease of the size of preneoplastic nodules. Similarly, Sun et al. [29] demonstrated that autophagy inhibition by CQ in the tumor-forming stage of DENA-induced HCC, remarkably reduced tumor growth by decreasing cell survival and proliferation. Moreover, it has been also reported that the combination of sorafenib with chloroquine produced more pronounced tumor suppression in HCC, both *in vivo* and *in vitro* [33]. In further support of the hypothesis that inhibition of autophagy plays an anti-tumoral effect in the early stages of the carcinogenic process, Amiodarone increases the percentage of KRT-19^+^ nodules, endowed with a higher proliferative capacity. By contrast, inhibition of autophagy by CQ resulted in an almost complete loss of nodules positive for this marker. These results, in turn, suggest a link between autophagy and the accumulation of KRT-19. Indeed, we found that the most aggressive KRT-19-positive nodules express high level of Ambra1 that, through its interaction with the protein phosphatase PP2A, regulates the stability of the oncoprotein and pro-mitotic factor c-Myc [8]. Thus, the expression of Ambra1 can both potentiates autophagy through its interaction with Beclin1 and VPS34, as well as favours the proliferation of the most aggressive nodules by binding to PP2A.

In conclusion, the present work raised the interesting hypothesis that autophagy in the liver may act as an homeostatic mechanism limiting the progression of preneoplastic cells to a more malignant stage. The finding that the administration of chloroquine is able to rapidly reduce the size of the KRT-19+ preneoplastic lesions suggests that the pharmacological modulation of autophagy may represent a possible therapeutic approach to interfere with the development of HCC.

## MATERIALS AND METHODS

### Animals and treatment

Male Fischer rats were obtained from Charles River (Milano, Italy). Guidelines for Care and Use of Laboratory Animals were followed during the investigation. All animal procedures were approved by the Ethical Commission of the University of Cagliari and the Italian Ministry of Health. Animals were treated with a single dose of diethylnitrosamine (DENA, 150 mg/kg) and, two weeks later, were subjected to the R-H protocol, consisting of a 2 week-diet supplemented with 0.02% 2-acetylaminofluorene (2-AAF) and a two/third partial hepatectomy (PH) [19]. Rats were then switched to basal diet all throughout the experiment and sacrificed 10 weeks after DENA administration (See Supp. Figure 1A.)

Another group of rats exposed to R-H protocol was given four doses of Chloroquine (50 mg/kg, Sigma-Aldrich, C6628) or Amiodarone (30 mg/kg, Sigma-Aldrich, A8423) starting 2 weeks after 2-AAF withdrawal (6 weeks after treatment with DENA). Rats were sacrificed 7 days after the first dose (See Supp. Figure 1B and 1C). BrdU was given in drinking water (1mg/1ml) for 5 days before the sacrifice.

### Histology and immunohistochemistry

Liver sections were fixed in 10% formalin and included in paraffin or quickly frozen by immersion in liquid nitrogen and processed for hematoxylin-eosin, or GST-P and KRT-19 immunohistochemistry, as described [21]. We considered as KRT-19 positive, all those lesions exhibiting a KRT-19 positive area of at least 5% of the total area of the preneoplastic lesion. The average area occupied by KRT-19 positive hepatocytes was at least 20% of the total area of the nodules microdissected for further analyses. Paraffin-embedded liver sections were used for p62, Ambra and Beclin 1 immunohistochemistry. Sections, were deparaffinized in xylene, incubated for 5 min each in 100%, 90%, 70%, and 50% ethanol for rehydration and immersed in 10 mM sodium citrate, pH 6.0, and microwaved for antigen retrieval. Endogenous peroxidase activity was blocked by 3% H_2_O_2_ for 5 min. After rinsing in phosphate-saline buffer (PBS) nonspecific antibody binding was reduced by incubating the sections with normal goat serum for 5 min. Sections were washed in PBS/1% BSA buffer and incubated with primary antibodies: rabbit anti-p62/SQSTM1 from MBL (Woburn, MA, USA) 1:400, rabbit anti AMBRA1 (ProSci) 1:100 and rabbit anti-BECN1 Antibody (Santa Cruz) 1:50 were used. Reactions were visualized using a streptavidin-biotin-immunoperoxidase system with DAB (Biogenex, San Ramon, CA) as chromogen substrates. Negative control staining was performed by omitting the primary antibody. Sections were counterstained in Mayer's acid hemalum.

### RNA extraction and qRT-PCR

Total RNA was extracted from preneoplastic lesions with the MirVana kit (Life Technologies) and stored at −80°C until needed. While RNA quantity was measured by NanoDrop ND1000 (Thermo Scientific), RNA integrity was assessed by Agilent Bioanalyzer 2100. Only RNA samples with a RIN (RNA Integrity Number) ≥ 7 were included in the study. RNA was retrotranscribed with High Capacity cDNA Reverse Transcription Kit (Life Technologies) using random primers. The expression levels of the examined genes were evaluated by Real-Time PCR analysis with an ABI PRISM 7300HT thermocycler (Life Technologies) on 6 samples of GST-P-positive preneoplastic nodules. All samples were run in triplicate. For qRT-PCR, The Power SYBR Green PCR Master Mix (Life Technologies) was used. The complete list of primer sequences used is listed in Supp. Table 1. After checking the specificity of the PCR products with the melting curve, data were then normalized to GAPDH expression and the expression level of different targets was calculated by 2^−ΔΔCt^. Analysis of LC3 expression was performed using specific TaqMan probes (Life Technologies) and GAPDH as endogenous control.

### Analysis of MicroRNA-224

cDNA was synthesized using the TaqMan MicroRNA Reverse Transcription Kit (Life Technologies) in accordance with the manufacturer's instructions. qRT-PCR amplification was performed with the reverse transcription product, TaqMan 2X Universal PCR Master Mix, No AmpErase UNG, miRNA-224 primers and probe mix (Life Technologies). The endogenous control 4.5S RNA(H) was used to normalize miRNA expression levels.

### Laser capture microdissection

GST-P^+^/KRT-19^+^ nodules were identified by immunohistochemical staining of 6μm-thick frozen liver sections. Nodules microdissection was done on 16μm serial sections with a Leica LMD6000, as previously described [21].

### Electron microscopy

Tissue samples were fixed with 2.5% glutaraldehyde (Assing Spa, R1012) in 0.1 M cacodylate buffer for 1 h at 4°C (sodium cacodylate trihydrate, Sigma-Aldrich, C4945), and postfixed in 1% osmium tetroxide (Sigma-Aldrich, 75632) in 0.1 M cacodylate buffer for 1 h. Samples were then dehydrated in graded ethanol and embedded in Epon resin (AGAR 100, Agar Scientific R1045). Ultrathin sections were stained with 2% uranyl acetate (Sigma-Aldrich, 73943) and observed under a Zeiss EM900 transmission electron microscope. Images were captured digitally with a Mega View II digital camera (SIS; Zeiss).

### Statistics

Data are expressed as mean ± standard deviation (SD) or mean ± standard error (SEM). Analysis of significance was done by t Student's test using the GraphPad software (La Jolla, California).

### SUPPLEMENTARY MATERIAL TABLE AND FIGURE



## Abbreviations

2-AAF: 2-acetylaminofluorene
Atg: autophagy-related genes
BrdU: 5-bromo-2′-deoxyuridine
CQ: chloroquine
DENA: diethylnitrosamine
GAPDH: glyceraldehyde 3-phosphate dehydrogenase
GST-P: placental glutathione S-transferase
HBV: hepatitis B virus
HCC: hepatocellular carcinoma
IHC: immunohistochemistry
KRT-19: cytokeratin-19
PH: partial hepatectomy
qRT-PCR: quantitative reverse transcriptase polymerase chain reaction
R-H model: Resistant-Hepatocyte model.
