# Supplementary material for: Induction of autophagy promotes the growth of early preneoplastic rat liver nodules
Source: Oncotarget. 2015 Dec 31;7(5):5788–99. doi: 10.18632/oncotarget.6810 (PMC4868721; doi:10.18632/oncotarget.6810)
Supplement: Supplementary file 1 [file oncotarget-07-5788-s001.pdf]

Induction of autophagy promotes the growth of early preneoplastic rat liver nodules

Supplementary Material

Supp. Table 1. Primers used for qRT-PCR analysis.

Gene Symbol	Forward Primer	Reverse Primer
**Ulk1**	GGCTTACAGACTGCCATTGA	GATACCACGCTGGCCTTATAC
**Ambra1**	ATACTACGCCCAGAGGATGA	GAAGAAGAGGAGGTGGAAGAAC
**Beclin1**	CAGGAACTCACAGCTCCATTAC	CCATCCTGGCGAGTTTCAATA
**Atg12**	GCCTCGGAGCAGTTGTTTA	ATGTAGGACCAGTTTACCATCAC
**Atg5**	TCCAACGTGCTTTACTCTCTATC	TGTCAGTTACCAGCGTCAAATA
**p62**	CTAGGCATCGAGGTTGACATT	CTTGGCTGAGTACCACTCTTATC
**FoxO3**	CCCAACCAGCTCCTTTAACA	GTAGTGCGACACGGAAGAAA
**Gapdh**	ACTCCCATTCTTCCACCTTTG	CCCTGTTGCTGTAGCCATATT

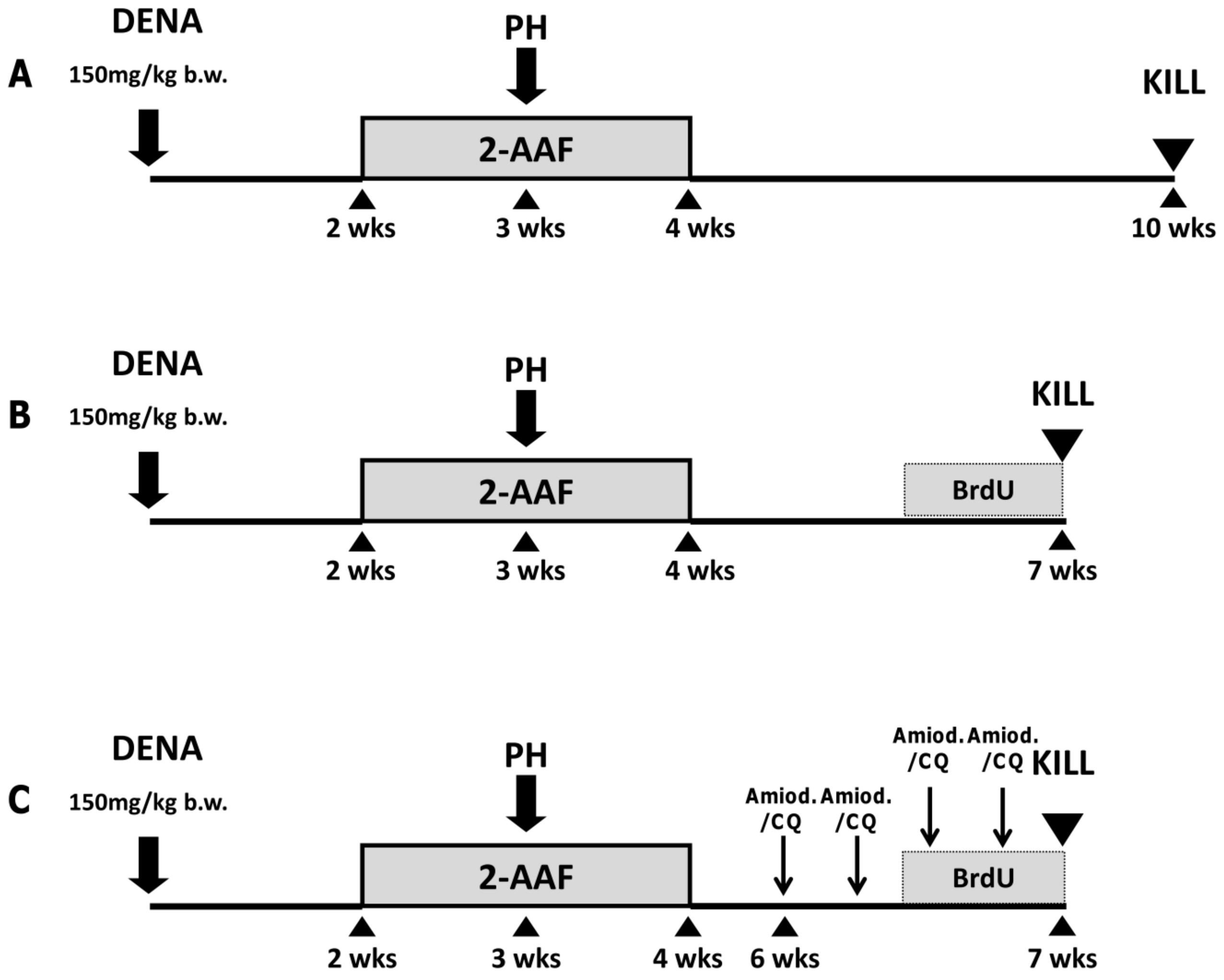

Supp. Figure 1 :
